# Clinical impact of the perioperative management of oral anticoagulants in bleeding after colonic endoscopic mucosal resection

**DOI:** 10.1186/s12876-019-1124-8

**Published:** 2019-12-02

**Authors:** Shoko Ono, Marin Ishikawa, Kana Matsuda, Momoko Tsuda, Keiko Yamamoto, Yuichi Shimizu, Naoya Sakamoto

**Affiliations:** 1 Division of Endoscopy, Hokkaido University Hospital, Nishi-7, Kita-15, Kita-ku, Sapporo, 060-8638 Japan; 2 Department of Gastroenterology, Graduate School of Medicine and Faculty of Medicine Hokkaido University, Sapporo, Japan

**Keywords:** Warfarin, Direct oral anticoagulant, Heparin bridging therapy, Endoscopic mucosal resection, Colon polyp

## Abstract

**Background:**

Heparin bridging therapy (HBT) is indeed related to a high frequency of bleeding after endoscopic mucosal resection (EMR). In this study, our aim was to investigate clinical impact of management of oral anticoagulants without HBT in bleeding after colonic EMR.

**Methods:**

From data for patients who underwent consecutive colonic EMR, the relationships of patient factors and procedural factors with the risk of bleeding were analysed. Our management of antithrombotic agents was based on the shortest cessation as follows: the administration of warfarin was generally continued within the therapeutic range, and direct oral anticoagulants (DOACs) were not administered on the day of the procedure. We calculated bleeding risks after EMR in patients who used antithrombotic agents and evaluated whether perioperative management of anticoagulants without HBT was beneficial for bleeding.

**Results:**

A total of 1734 polyps in 825 EMRs were analysed. Bleeding occurred in 4.0% of the patients and 1.9% of the polyps. The odds ratios for bleeding using multivariate logistic regression analysis were 3.67 in patients who used anticoagulants and 4.95 in patients who used both anticoagulants and antiplatelet agents. In patients with one-day skip of DOACs, bleeding occurred in 6.5% of the polyps, and there were no significant differences in bleeding risk between HBT and continuous warfarin or one-day skip DOACs.

**Conclusions:**

The use of oral anticoagulants was related to bleeding after colonic EMR, and perioperative management of oral anticoagulants based on the shortest cessation without HBT would be clinically acceptable.

## Background

Colonic polypectomy and endoscopic mucosal resection (EMR) are standard procedures for the treatment of colon polyps; however, these procedures have a risk of bleeding. Previous studies have shown that post-polypectomy bleeding (PPB) occurred in several patients, and some risk factors of PPB were shown [1–3]. Kim et al. reported that the following factors (old age, comorbid cardiovascular or chronic renal disease, anticoagulant use, polyp size greater than 1 cm, gross morphology of polyps, such as pedunculated polyps, or laterally spreading tumours, poorer bowel preparation, cutting mode of the electrosurgical current and the inadvertent cutting of a polyp) were associated with PPB from a multicentre prospective, cross-sectional study [2]. In addition, Shalman et al. reported that clopidogrel and warfarin should be discontinued during the periprocedural period to prevent the occurrence of PPB from a systematic review with a meta-analysis; however, there were no data about direct oral anticoagulants (DOACs) [3].

Anticoagulant agents prevent thrombotic events in patients with arterial fibrillation (Af) and venous thromboembolism, including pulmonary thromboembolism and deep vein thrombosis [4–6]. Recently, the prescription of DOACs has increased because the clinical management of DOACs is easier than that of warfarin [7]. In fact, there have also been recent reports on perioperative events in endoscopic resection for patients receiving DOACs [8]. Additionally, heparin bridging therapy (HBT), which has been commonly used for the perioperative management of warfarin, is problematic in bleeding and clinical practice. However, the relationship between the perioperative management of anticoagulants and delayed bleeding has not been clarified, and there have been little real-world data on DOACs as new options. We therefore investigated bleeding risk after colonic EMR in patients taking oral coagulants and evaluated whether the continuous use of warfarin and the one-day skip of DOACs could be eligible for perioperative management without HBT.

## Methods

### Patients

The data for consecutive patients who received colonic EMR at Hokkaido University Hospital during the period from January 2013 to May 2017 were retrospectively analysed. Patients who received endoscopic submucosal dissection (ESD) and cold polypectomy (resection without electrocautery device) were excluded. The patients were classified according to taking antithrombotic agents (only antiplatelets, only anticoagulants and both antiplatelets and anticoagulants), and the patients who did not use anticoagulants or antiplatelets were analysed as controls.

Permission to study patient records was given by the Hokkaido University Hospital Review Board (017–0153; approved on October 6, 2017).

### Perioperative management of antithrombotic agents

Before planning EMR, we consulted with the prescribing doctors about how to manage antithrombotic agents, including the need for HBT. Generally, the period for withdrawal of antiplatelet agents was in accordance with the Japan Gastroenterological Endoscopy Society (JGES) guidelines published in 2012 [9]. For patients with a low thrombotic risk, the administration of aspirin (ASA) and thienopyridine was discontinued for 3 days and 5 days, respectively. The administration of other agents was stopped at 1 day before the procedure. For patients with a high thrombotic risk who were taking multiple antiplatelets, monotherapy with ASA or cilostazol was continued.

**Fig. 1:**
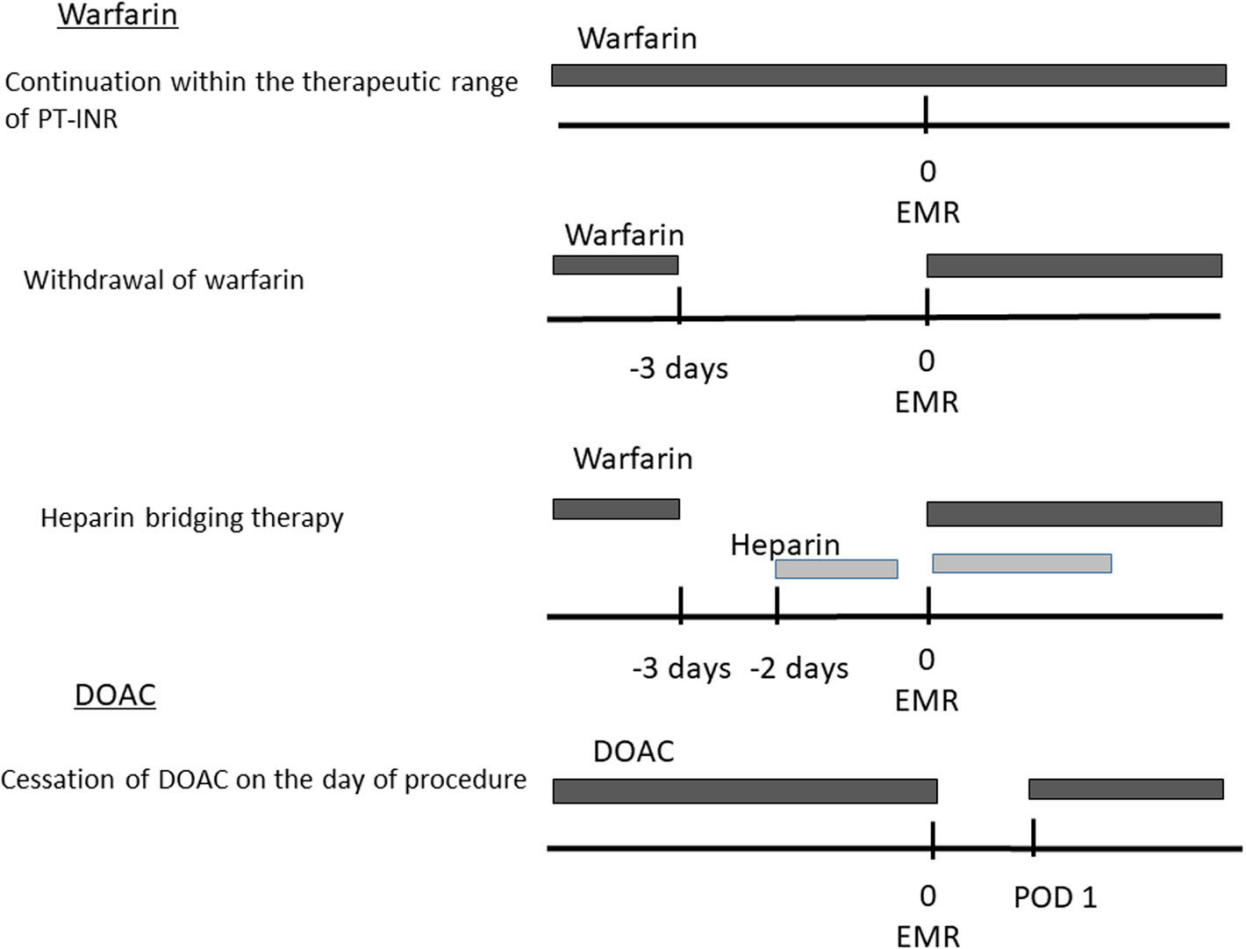
Management of anticoagulants. The administration of warfarin is generally continued within the therapeutic range of the prothrombin time-international normalized ratio (PT-INR). For patients taking warfarin with low-thromboembolic risk, 3 days withdrawal of warfarin is performed. In heparin bridging therapy, the administration of warfarin is discontinued for 3 days, and heparin is intravenously administered for 2 days. The administration of heparin is stopped 4–6 h before the procedure and is immediately restarted with warfarin after surgery. A direct oral anticoagulant (DOAC) is orally administered on the day before the procedure, and administration is restarted on the morning of postoperative day (POD) 1

Our management of anticoagulants is shown in Fig. 1. For patients taking warfarin who were judged by the prescribing doctor to have high-risk thromboembolism, HBT or the continuous use of warfarin were selected by the prescribing doctor. HBT was conducted as follows: the administration of warfarin was discontinued for at 3 days before EMR and unfractionated heparin (UFH) was intravenously administered for 2 days. Administration of heparin was stopped at 4–6 h before the procedure and was immediately restarted with warfarin after EMR [10]. The continuous use of warfarin was conducted within the therapeutic range of the prothrombin time (PT)-international normalized ratio (INR) (less than 2.6). For patients taking warfarin with low-thromboembolic risk, 3 days withdrawal of warfarin was performed. The administration of DOACs (dabigatran, apixaban, edoxaban and rivaroxaban) was generally stopped only on the day of EMR, and administration was restarted on the morning of postoperative day (POD) 1.

For users of anticoagulants, PT (Thromborel S (Siemens Healthcare Diagnostics, Marburg, Germany)) and activated partial thromboplastin time (APTT) (Thrombocheck aPTT-SLA (Sysmex, Kobe, Japan)) were checked immediately before EMR for confirmation of appropriate values. All patients provided written informed consent to the perioperative management of antithrombotic agents. The last date for taking an antithrombotic drug for each patient was checked and recorded immediately before the procedures by nurses.

### Endoscopic resection

EMR by the standard methods was performed for lesions that could be snared. Saline for submucosal injection with a Rotatable Snare (Boston Scientific Corporation, Tokyo, Japan) and a high-frequency electrosurgical generator ICC200 or VIO300D (ERBE Elektromedizin GmbH, Germany) were generally used. The use of a clip for prophylaxis of bleeding depended on the operator. Complete haemostasis was confirmed after resection. All patients provided written informed consent to undergo the proposed EMR.

### Definition of bleeding after EMR

Bleeding was defined as a confirmation of active bleeding or coagula on the iatrogenic ulcer by emergent endoscopy within 30 days after EMR. When haematochezia or a decrease in haemoglobin level (more than 2 g/dl) was observed after EMR, emergent colonoscopy was immediately performed.

### Measured outcomes

In this retrospective study, the medical records were reviewed for the following patient factors: age, sex, blood pressure (before EMR), underlying disease, use of antithrombotic agents (anticoagulants and antiplatelets), replacement of heparin and laboratory data. The location, size and morphology of the polyps, prophylactic clip closure, histology and operator of EMR were also analysed as procedural factors.

### Statistical analysis

JMP® Pro 11.2.0 (SAS Institute Inc.) was used for data analysis. Summarized numerical data were expressed as medians with standard deviations. Categorical data were compared using the χ^2^ test, and numerical data were compared using Student’s t-test. Multivariate logistic regression analysis was performed for calculating odds ratios (ORs) of bleeding. A *p* value of < .05 in each analysis was considered statistically significant.

## Results

### Patient factors and procedural factors related to bleeding after EMR

**Fig. 2:**
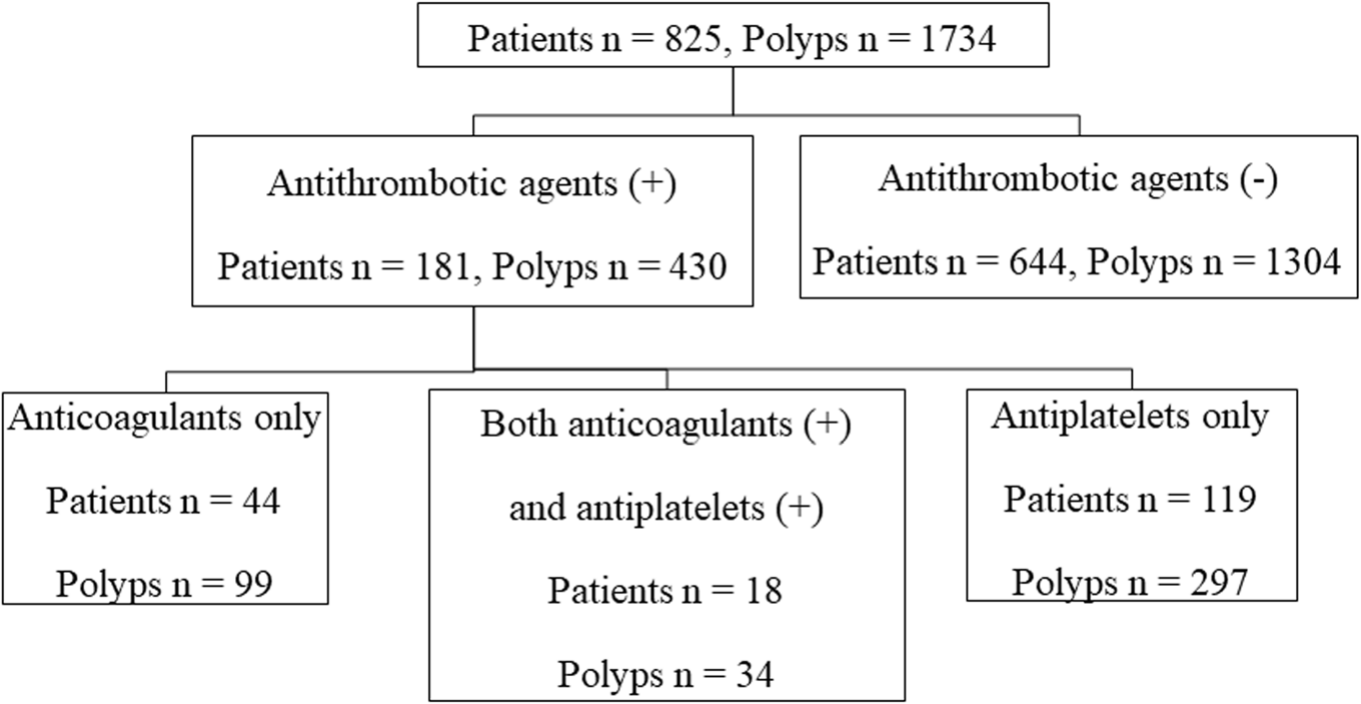
Use of antithrombotic agents in the subjects. A total of 181 patients (21.9%) used antithrombotic agents, 44 patients used only oral anticoagulants, and 18 patients used both anticoagulants and antiplatelets

During the study period, 1734 polyps in 825 patients were resected. The use of antithrombotic agents in the subjects is shown in Fig. 2. For anticoagulants, 34 patients used warfarin, and 28 patients used DOACs (rivaroxaban *n* = 10, apixaban *n* = 9, edoxaban *n* = 5, dabigatran *n* = 4).

Bleeding occurred in 33 patients (4%) and 33 polyps (1.9%). Patient factors (per patient) and procedural factors (per polyp) were compared in patients with bleeding and those without bleeding (Tables 1 and 2). Patients who had taken anticoagulants or both anticoagulants and antiplatelets had significant risks of bleeding.

**Table 1:** Patient factors in bleeding after colonic endoscopic mucosal resection

	Bleeding (+), *n* = 33	Bleeding (−), *n* = 792	*p*
Age, mean ± SD, years	63.6 ± 9.26	65.5 ± 10.9	0.323
Males/ Females, n	24/ 9	478/ 314	0.143
Underlying disease, *n* (%)			
Diabetes mellitus	6 (18.2)	160 (20.2)	0.774
Cerebral infarction	0	39 (4.9)	0.707
Arrhythmia	5 (15.2)	64 (8.1)	0.191
Ischemic heart disease	7 (21.2)	64 (8.1)	0.023
Chronic renal failure	3 (9.1)	22 (2.8)	0.089
Antithrombotic agents, *n* (%)	12 (36.4)	169 (21.3)	0.054
Antiplatelets	7 (21.2)	130 (16.4)	0.484
Anticoagulants	8 (24.2)	54 (6.8)	0.002
Both of drugs	3 (9.1)	15 (1.9)	0.034
Systolic blood pressure,	133.7 ± 25.0	134.5 ± 20.5	0.818
mean ± SD, mmHg			
PT, mean ± SD, seconds	14.23 ± 4.65	12.75 ± 8.68	0.486
APTT, mean ± SD, seconds	32.68 ± 6.04	29.89 ± 4.47	0.011

**Table 2:** Procedural factors in bleeding after colonic endoscopic mucosal resection

	Bleeding (+), *n* = 33	Bleeding (−), *n* = 1701	*p*
Location, right/ left/ rectum	14/ 12/ 7	846/ 639/ 216	0.385
Morphology, pedunculated, n	10	221	
(%)	(30.3)	(13.0)	0.004
Size, mean ± SD, mm	14.4 ± 8.02	9.26 ± 5.66	< 0.001
Prophylactic clip, *n* (%)	21 (63.6)	917 (53.9)	0.274
Histological cancer	4	111	0.250
component, *n* (%)	(12.1)	(6.5)	
Procedure by expert^a^, *n* (%)	12 (36.4)	509 (29.9)	0.432

^a^Endoscopist having experience more than10 years

### Bleeding according to antithrombotic agents

Table 3 shows bleeding rates and risk per polyp after colonic EMR for patients who took antithrombotic agents. The use of anticoagulants during the perioperative period with or without antiplatelets was associated with a high bleeding risk; furthermore, HBT showed the highest risk for bleeding (Bleeding rates; 7.14, 5.48 and 9.68%, Odds ratios 4.95, 3.67 and 6.88, respectively).

**Table 3:** Bleeding rates and risk after colonic endoscopic mucosal resection according to antithrombotic agents

	None	Antiplatelets	Anticoagulants	Antiplatelets and anticoagulants	HBT^a^
Polyp, n	1304	297	74	28	31
Size, mean ± SD, mm	9.5 ± 5.9	8.6 ± 4.9^b^	8.8 ± 6.0	8.8 ± 4.2	9.2 ± 5.1
Morphology	187	33	7	1	3
pedunculated, *n* (%)	(14.3)	(11.1)	(9.5)	(3.6)	(9.7)
Bleeding, *n* (%)	20 (1.53)	4 (1.35)	4 (5.48)	2 (7.14)	3 (9.68)
Odds	1	0.88	3.67	4.95	6.88
(95%CI)		(0.30–2.58)	(1.22–11.0)	(1.10–22.2)	(1.93–24.5)
*p*	–	0.81	0.021	0.038	0.020

^a^Heparin bridging therapy, ^b^None: Antiplatelets, *p* < 0.05

### Bleeding according to actual management of anticoagulants

**Fig. 3:**
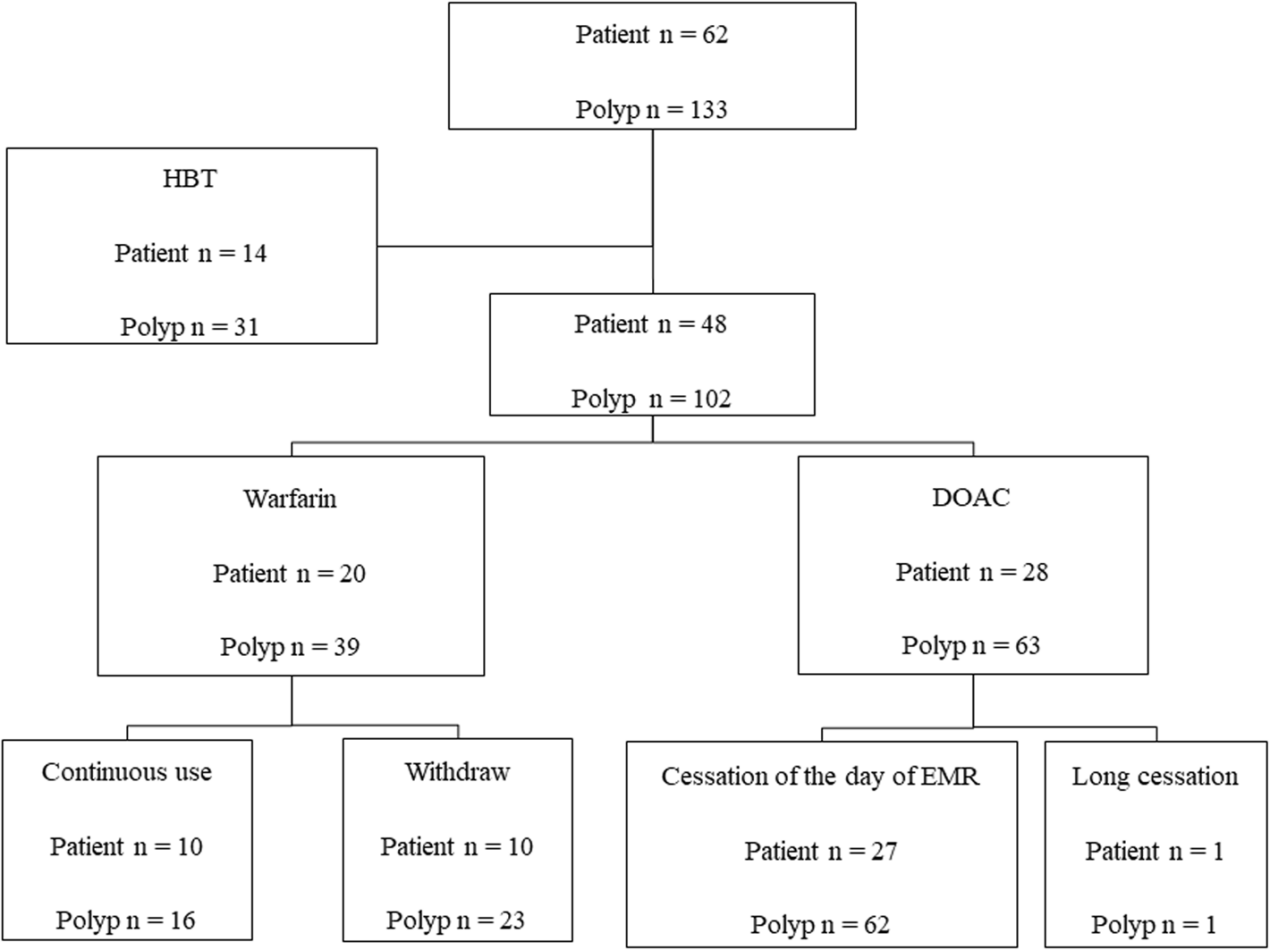
Actual withdrawal or continuation of anticoagulants before endoscopic mucosal resection. Heparin bridging therapy (HBT) was conducted in 14 patients who were administered anticoagulants. Warfarin was continuously administered in 10 patients during the perioperative period, and a direct oral anticoagulant (DOAC) was skipped only on the day of endoscopic mucosal resection (EMR) in 27 patients. The withdrawal of anticoagulants was confirmed by interviews with patients immediately before the EMR

Figure 3 shows actual withdrawal periods or the continuation of anticoagulants. The characteristics were compared among patients who complied with HBT, those with continuous use of warfarin and 1 day cessation of DOACs and patients who did not use antithrombotic agents as controls (Table 4). The bleeding rates in the HBT group and continuous DOACs group were significantly higher than those in the control group (*p* <  0.05), and high bleeding risk ratios were calculated (Table 5). However, there were no significant differences in bleeding risk between HBT and continuous warfarin or one-day skip DOACs (Odds ratios; 4.94, 3.29 and 4.94, respectively).

**Table 4:** Characteristics of each group according to the management of anticoagulants

	HBT^a^ (*n* = 14)	Continuous use of warfarin (*n* = 10)	One-day skip of DOAC (*n* = 28)	Control (*n* = 643)
Age, mean ± SD, years	61 ± 17.6	71.3 ± 11.5	69.6 ± 7.1	64.5 ± 11.0
Males/ Females, n	11/ 3	7/ 3	21/ 7	370/ 273
Using antiplatelets, *n* (%)	4 (28.6)	3 (30.0)	6 (21.4)	0
PT, mean ± SD, seconds	14.8 ± 2.1	22.1 ± 4.4^*, **^	12.9 ± 0.74	12.6 ± 9.46
APTT, mean ± SD, seconds	33.4 ± 4.3^*^	37.6 ± 3.7^*, ***^	34.2 ± 4.4^*^	29.4 ± 4.5
Polyp, n	31	16	62	1304
Pedunculated, *n* (%)	3 (9.7)	1 (6.3)	5 (8.1)	18 (1.4)
Size, mean ± SD, mm	10.4 ± 7.0	8.2 ± 6.2	8.9 ± 5.6	9.5 ± 5.9
Prophylactic clip, *n* (%)	21 (67.7)	9 (56.3)	34 (54.8)	688 (52.8)
Procedure by expert^b,^ *n* (%)	10 (32.6)	4 (25.0)	10 (16.1)	404 (31.0)

^a^*HBT* heparin bridging therapy, ^b^Endoscopist having experience more than10 years

^*^ *v. s.* Control, *p* < 0.05, ^**^ *v. s.* DOAC *p* < 0.05, ^***^ *v. s.* HBT *p* < 0.05

**Table 5:** Bleeding after colonic endoscopic mucosal resection in the management of continuous use of warfarin and one-day skip of DOAC

Bleeding	HBT^a^ (*n* = 14)	Continuous use of warfarin (*n* = 10)	One-day skip of DOAC (*n* = 27)	Control (*n* = 643)
n	3	1	4	20
Per-patient, %	21.4^*^	10.0	14.8^*^	3.1
Per polyp, %	9.7^*^	6.3	6.5^*^	1.5
Odds	4.94	3.29	4.94	1
(95%CI)	(1.04–23.5)	(0.40–27.2)	(1.57–15.5)	
*p*	0.045	0.27	0.0062	

^a^*HBT*heparin bridging therapy, ^*^ *v. s.* Control, *p* < 0.05

DOACs were administered on the morning of POD 1, and bleeding occurred in 4 patients on POD 2 (apixaban), POD 3 (rivaroxaban), POD 4 (dabigatran) and POD 9 (apixaban and ASA). On the other hand, bleeding occurred in the patient who continuously used warfarin on POD1 and in the patients who received HBT on POD1 and 5. Endoscopic haemostasis was successfully performed on those patients.

No thrombotic events occurred at 1 month after the procedures.

## Discussion

The updated guidelines of the American Society for Gastrointestinal Endoscopy (ASGE) and the European Society of Gastrointestinal Endoscopy (ESGE) in 2016 recommend the cessation of warfarin for 5 days during endoscopic surgery in patients with a low thrombotic risk [11, 12]. However, both guidelines recommended HBT instead of warfarin in patients with a high thrombotic risk during the perioperative period.

Regarding DOACs, cessation for 1 to 3 days is recommended in the ASGE guidelines, and cessation for at least 2 days is recommended in the ESGE guidelines before endoscopic surgery. DOACs are re-administered after confirmation of haemostasis, and anticoagulant therapy is therefore stopped for more than several days.

Since the interruption of the administration of anticoagulants during the perioperative period can cause serious thrombotic events, Japanese guidelines from the JGES edited in 2012 recommended HBT in patients who are scheduled to undergo endoscopic resection with a high bleeding risk [9]. However, the management of HBT is complicated, and there have been some reports on the risk of delayed bleeding showing rates of more than 10–20% after endoscopic resection [10, 13]. Therefore, the JGES updated the guidelines regarding the use of anticoagulants in 2017. The continuous use of warfarin in the therapeutic range of the PT-INR and the one-day skip of DOACs, equivalent to HBT, are now recommended for management during the perioperative period [14]. For warfarin users, continuous warfarin might be a better management than HBT for both bleeding and thromboembolism. Unfortunately, there were no significant differences in delayed bleeding after colonic EMR in this study because of the small sample size.

The strongest point in this study is that the actual cessation periods of anticoagulants were investigated. Patients often forget about the cessation of drugs or tend to prolong the cessation periods by themselves. In addition, the instructions for the cessation of anticoagulants are not constant and depend on individual doctors. Bleeding risk after endoscopic procedures depends on the kind and number of drugs are used and whether the drugs are discontinued or continued during the perioperative period. However, when discussing the bleeding risk of antithrombotic drugs, few studies have confirmed drug compliance.

There have been a few reports on the effects of DOACs on endoscopic procedures. When DOACs were not administered only on the day of EMR based on their short half-lives, the bleeding rate after colonic EMR in our study was 6.5%, which is almost as high as that with HBT. DOACs have maximum effect in 2–4 h after taking drugs, therefore DOAC users are equally high risk of bleeding with the patients who continuously use warfarin. Although DOACs do not require frequent monitoring of their anticoagulation effect, the risks of bleeding and hypercoagulability were investigated by using molecular markers in some studies [15]. Peak PT values were significantly more prolonged than trough PT values, and prolonged peak PT (≥ 20 s) increased the risk of bleeding in Japanese patients with non-valvular Af receiving rivaroxaban [16]. None of our patients with delayed bleeding who were taking DOACs had PT prolonged for more than 20 s. DOACs are related to GI bleeding, and it has been hypothesized that non-absorbed, active anticoagulant agents within the GI tract cause bleeding of vulnerable mucosal breaks [17].

The incidence rates of thrombotic events related to endoscopic surgery were 0–4.2% [18, 19]. In addition, the interruption of warfarin for 4 to 7 days induced thromboembolic events in approximately 1% of patients [20]. Fortunately, embolic events did not occur in our patients. There are several limitations in this study. First, this study was a retrospective study at a single institution with a small sample size. Furthermore, the superiority of the perioperative management of oral anticoagulants without HBT was not shown, and further prospective studies are therefore needed to establish the safe perioperative management of oral anticoagulants in patients undergoing colonic EMR.

## Conclusion

The use of oral anticoagulants was related to bleeding after colonic EMR, and perioperative management of oral anticoagulants based on the shortest cessation without HBT would be clinically acceptable.

## Data Availability

All data generated or analyzed during this study are included in this published article.

## Abbreviations

Af: Arterial fibrillation
APTT: Activated partial thromboplastin time
ASA: Aspirin
ASGE: American Society for Gastrointestinal Endoscopy
CIs: Confidence intervals
DOACs: Direct oral anticoagulants
EMR: Colonic polypectomy and endoscopic mucosal resection
ESD: Endoscopic submucosal dissection
ESGE: European Society of Gastrointestinal Endoscopy
HBT: Heparin bridging therapy
JGES: Japan Gastroenterological Endoscopy Society
ORs: Odds ratios
POD: Postoperative day
PPB: Post-polypectomy bleeding
PT-INR: Prothrombin time-international normalized ratio
UFH: Unfractionated heparin
